# Gold Nanostars Bioconjugation for Selective Targeting and SERS Detection of Biofluids

**DOI:** 10.3390/nano11030665

**Published:** 2021-03-08

**Authors:** Caterina Dallari, Claudia Capitini, Martino Calamai, Andrea Trabocchi, Francesco Saverio Pavone, Caterina Credi

**Affiliations:** 1 European Laboratory for non-linear Spectroscopy (LENS), University of Florence, 50019 Sesto Fiorentino, Florence, Italy; capitini@lens.unifi.it (C.C.); calamai@lens.unifi.it (M.C.); francesco.pavone@unifi.it (F.S.P.); 2 Department of Physics, University of Florence, 50019 Sesto Fiorentino, Florence, Italy; 3 National Institute of Optics-National Research Council (CNR-INO), 50019 Sesto Fiorentino, Florence, Italy; 4 Department of Chemistry “Ugo Schiff”, University of Florence, 50019 Sesto Fiorentino, Florence, Italy; andrea.trabocchi@unifi.it

**Keywords:** gold nanoparticles, gold nanostars, surface-enhanced Raman spectroscopy (SERS), bioconjugation, neuroblastoma cells, hemagglutinin, biofluids

## Abstract

Gold nanoparticles (AuNPs) show physicochemical and optical functionalities that are of great interest for spectroscopy-based detection techniques, and especially for surface enhanced Raman spectroscopy (SERS), which is capable of providing detailed information on the molecular content of analysed samples. Moreover, the introduction of different moieties combines the interesting plasmonic properties of the AuNPs with the specific and selective recognition capabilities of the antibodies (Ab) towards antigens. The conjugation of biomolecules to gold nanoparticles (AuNPs) has received considerable attention for analysis of liquid samples and in particular biological fluids (biofluids) in clinical diagnostic and therapeutic field. To date, gold nanostars (AuNSts) are gaining more and more attention as optimal enhancers for SERS signals due to the presence of sharp branches protruding from the core, providing a huge number of “hot spots”. To this end, we focused our attention on the design, optimization, and deep characterization of a bottom up-process for (i) AuNPs increasing stabilization in high ionic strength buffer, (ii) covalent conjugation with antibodies, while (iii) retaining the biofunctionality to specific tag analyte within the biofluids. In this work, a SERS-based substrate was developed for the recognition of a short fragment (HA) of the hemagglutinin protein, which is the major viral antigen inducing a neutralizing antibody response. The activity and specific targeting with high selectivity of the Ab-AuNPs was successfully tested in transfected neuroblastoma cells cultures. Then, SERS capabilities were assessed measuring Raman spectra of HA solution, thus opening interesting perspective for the development of novel versatile highly sensitive biofluids sensors.

## 1. Introduction

Antibody-gold nanoparticle (Ab-AuNP) bioconjugates are widely exploited in the field of optical biosensing. AuNPs are experiencing large interest due to their excellent optical properties related to localised surface plasmon resonance (LSPR), which allows various signal transduction options [1]. Moreover, the introduction of different moieties on the surface of NPs combines the interesting plasmonic properties with the selective and specific recognition capabilities of the Ab towards antigens [2]. Thus, it becomes straightforward the possibility to achieve reliable and reproducible methods of conjugation to develop platforms for next generation technologies ranging from diagnostics to therapeutics [3]. The two main strategies applied for the conjugation of antibodies to AuNPs are (i) physisorption and (ii) chemisorption [4]. The former method is the simplest, since it does not require surface chemistry modification but simply relies on electrostatic interactions, hydrogen bonding, hydrophobic interactions, and van der Waals forces between AuNPs and Ab. Despite its easiness, this method leads to random orientation and a very low quantity of Ab adsorbed on the NPs’ surface [5]. Further, a method based on ionic adsorption requires pH values close to the isoelectric point of the Ab, drastically lowering stability at different pH conditions [6]. Chemisorption is more challenging than physisorption, but it usually provides a higher stability of bioconjugates, improving the accessibility of epitope as well as the reproducibility of the binding events. Covalent coupling is advantageous with respect to physical adsorption as the linkage would involve the Fc region, leaving the antigen-binding site Fab region preserving its full function [7]. To this end, the surface of AuNPs can be modified with various heterobifunctional ligands containing thiols for grafting to the particles and functional pending groups, as end-chain moieties, for further covalent conjugation of antibodies, ensuring high colloidal stability and accessibility.

The vast majority of works found in the literature focus on IgG-type antibodies attached to spherical AuNPs as the easiest shape of nanoparticles to be synthesized and modified [8,9,10]. More recently, it has been demonstrated that changing the shape from spheres to more anisotropic forms such as stars result in better optical performances, due to both intrinsic NP surface plasmons as well as on local field “hotspots”, mainly represented by tips with nanosized radius of the curvature [3]. As a matter of fact, some works on the functionalization of multibranched Au nanostars (AuNSts) were already published. Li-Kai Lin et al. [11] developed a bisphenol A (BPA) detection method, coating AuNSts with anti-BPA antibody as a recognition system, exploiting electrostatic interactions to physisorb antibodies to gold surface. Other works based on physical adsorption on AuNSts were reported in the literature [12,13]. In this context, Oliveira M. J. et al. published a really interesting and extensive work on the characterization of bioconjugation process through non-covalent interactions of antibodies with bifunctional ligands grafted onto the surface of AuNSts [14]. For what concern the chemisorption strategy instead, some articles can be found in literature. Liang S. et al. [15] as well as Tan H. et al. [16] attached antibodies onto PEG-modified AuNSts via carbodiimide chemistry. However, despite the presence of other works based on covalent linking of Ab to star-shaped particles, [17,18,19,20] to the best of our knowledge a deep characterization and quantification of such a process is still lacking, which is of fundamental importance for bioconjugate stability and retaining of the ability to recognize and to bind its corresponding antigen, when working with such highly complex systems as biofluids. 

Herein, we present an extensive study of AuNSts surface engineering based on the optimization and characterisation of (i) exchange ligand process with a bifunctional molecule in order to raise the stability of the colloidal nanoparticles solution, and (ii) further covalent bioconjugation with baits specific for the recognition of the analyte to be targeted in biological fluids. Biofluids like blood, urine, saliva, and cerebrospinal fluids are usually complex matrices characterized by a huge amount of proteins, lipids, and salts with a consequently high ionic strength. Electrostatic interactions between negative charges of citrate as ligand molecules and particles are strongly affected by factors like the ionic strength, pH of solution, temperature, and even time. Thus, a stabilizing agent which interacts in a strong covalent way with surface’s gold atoms of the NP was exploited to increment the stability of the final bioconjugate, ensuring a safe interaction with buffer solutions as well as with real biofluids [21]. It becomes a crucial point to have particles completely covered with surfactant which have the role to (i) avoid aggregation and formation of clusters and (ii) to provide a bio-friendly environment [22]. Such a deep study opens the way for a wide range of applications in the clinical field, exploiting the enhancing capabilities deriving from optical features of AuNPs combined with powerful characteristics coming from Raman spectroscopy. Raman allows the recognition of distinct molecules based on their vibrational bands, producing a univocal molecular fingerprint; moreover, it is a non-destructive as well as non-invasive technique and does not require sample preparation [23]. Chemical properties of AuNSts were tailored to be in resonance with the Raman system in order to (i) provide surface enhanced Raman scattering (SERS) effect and consequently to (ii) increase their chemical affinity towards the marker of interests and thus decreasing the relative distance between the SERS substrates and the targets itself [24]. 

In more details, native citrate molecules interacting with gold surface of NSts through electrostatic interaction were replaced by bifunctional polyethylene glycol (PEG) molecules, which covalently bind through the formation of thiol-gold bonds [25]. A huge number of studies on functionalization with PEG molecules of gold sphere-shaped particles are currently being published, however little is known about modification of multibranched star-shaped [21,26,27,28]. The complex morphology with high aspect ratio sharp tips could affect the homogeneity of functionalization and conjugation processes, and consequently could restrict reproducibility and applicability. The first studies conceived the optimization of the quantity of PEG required to reach stability in PBS buffer. To this end, PEG-modified NSts were characterized by ultraviolet-visible spectroscopy (UV-Vis), dynamic light scattering technique (DLS), zeta-potential measurements (ζ), and agarose gel electrophoresis (AGE). Then, a generic immunoglobulin G (IgG) antibody was conjugated to NSts via amide bonds formation through terminal carboxylic acid groups of PEG and primary amine groups of antibodies [2,29,30]. Covalent conjugation between antibodies and PEG-NSts was investigated through UV-Vis, DLS, ζ, while antibody coverage to AuNP can also be quantified from fluorescence measurements. Finally, to demonstrate the potentiality of developed bioconjugates as SERS-based substrates for liquid detection, PEG-NSts constructs were functionalized to selectively tag a short peptide (HA) from hemagglutinin, a homotrimeric glycoprotein found on the surface of influenza viruses, which is accountable for the first bond of the virion to the host cell surface, by identification of terminal sialic acid moieties and further fusion with the cell membrane [31,32]. Anti-hemagglutinin peptide antibodies (antiHA) were grafted on the NPs and the retaining of biofunctionality and selectivity respect to target analyte were tested with confocal fluorescence images. The improved ability to tag diluted HA peptide directly in liquid samples were also proved with Raman-SERS measurements, demonstrating the capability of detect the analyte protein only in the presence of constructed NP-Ab bioconjugate.

## 2. Experimental Section

### 2.1. Raw Materials

Gold(III) chloride trihydrate (HAuCl_4_ · 3H_2_O); trisodium citrate dihydrate (C_6_H_5_O_7_Na_3_ · 2H_2_O); L-(+)-ascorbic acid (AA); silver nitrate (AgNO_3_); hydrochloric acid (HCl); nitric acid (HNO_3_ – 70%); Poly(ethylene glycol) 2-mercaptoethyl acetic acid (SH-PEG-COOH, M_n_ 7500); N-(3-Dimethylaminopropyl)-N-ethylcarbodiimide hydrochloride (EDC); N-Hydroxysuccinimide (NHS); HA Tag Monoclonal Antibody (2-2.2.14) with and without DyLight 550 and HA Synthetic Peptide from ThermoFisher Scientific (Waltham, MA, USA); AlexaFluor488- labelled IgG antirabbit were purchased from ThermoFisher Scientific (Waltham, MA, USA); all organic solvents were purchased from Merck (Darmstadt, Germany) and used without further purification. DMEM/F12 medium, FBS, penicillin/streptomycin solution, Opti-MEM were purchased from ThermoFisher Scientific (Waltham, MA, USA). FuGENE^®^ HD Transfection Reagent was purchased from Promega Corporation (Madison, WI, USA).

### 2.2. Gold Nanoparticles Synthesis and Functionalization

Gold-nanoparticles (Au-NPs) were synthesized by the seeded-growth process described by Yuan et al. [33]. The seed solution of 15 nm-nanospheres (NSps) was prepared by adding 1.5 mL of 30 mM HAuCl_4_ · 3H_2_O (1%) to 48.5 mL of boiling and stirring MilliQ (stirring 7 position, 250 °C). After 10 s, 4.5 mL of 38.8 mM sodium citrate solution was added to the solution. Solution was stirred under heating for 15 min, and then stirred without heating for 30 min. For nanostars (NSts) synthesis 0.083 mL of 30 mM HAuCl_4_ · 3H_2_O (1% solution) was added to 9.917 mL of MilliQ 60 μL of 1 M HCl and 100 μL of the NSps solution were added to the solution. Then, 100 μL of 2 mM AgNO_3_ and 50 μL of 0.1 M ascorbic acid were added simultaneously. The solution was stirred for 30–60 s, while its colour turned from light red to dark grey (or blue). Immediately afterwards, NSts were centrifuged for 20 min at 2500 rpm and redispersed in 100 μL of distilled water. Later, SH-PEG-COOH was used in a ligand-exchange reaction to replace the citrate layer with PEG in both citrate-capped nanospheres (Cit-NSps) and nanostars (Cit-NSts). Au-NPs solution was diluted to 1 nM; different volumes of a 0.01 M aqueous solution of PEG was added to the solution and stirred at RT for 12 h to reach [PEG]/[AuNP] molar ratio ranging from 0 to 100,000. The concentration of NSps was determined according to formula reported by Liu et al. [34]. For star-shaped particles the method of De Puig et al. was used to calculate the same parameters [35]. Pegylated-nanoparticles (PEG-NPs) were centrifuged at 25 °C—10,000 rpm for NSps and 2500 for NSts—for 20 min and redispersed in milliQ water.

### 2.3. Preparation of NP-Ab Bioconjugate

NP-Ab bioconjugates were prepared with 5 nM solution of the previously PEG functionalized nanoparticles. Incubation was in 0.1 M phosphate saline buffer (PBS) at a pH of 7.4 for 12 h at room temperature after activation with 0.4 mM EDC and 0.1 mM NHS (final concentration). An amount of 1 µL of 2 mg/mL stock solution of antibody was added to 100 µL of 5 nM PEG-NSts, resulting in a 10 times-dilution (20 µg/mL antibodies solution). Samples were then centrifuged for 15 min at 4 °C and 10,000 rpm for NSps and 2500 rpm for NSts. The supernatant was recentrifuged, pellets reunited and dispersed in 100 μL of PBS buffer with BSA 0.5% *w*/*v*. For cells incubation, pellets were dispersed in 100 μL of Leibovitz buffer with 3% FBS and 0.5% BSA. Protocol was applied to HA Tag Monoclonal Antibody with and without DyLight550 to test specificity, selective targeting, and capabilities of detection and to IgG-Alexa488 labelled to quantify the number of antibodies attached per particle. 

### 2.4. Cell Cultures and Transfection

Human SH-SY5Y neuroblastoma cells (A.T.C.C. Manassas, VA, USA) were cultured in Dulbecco’s Modified Eagle’s Medium (DMEM) (ThermoFisher Scientific, Waltham, MA, USA) F-12 supplemented with 10% FBS, 1% penicillin/streptomycin solution. Cells were cultured in a humidified chamber at 5% CO_2_ and 37 °C and grown until they reached 90% confluence. For transfection, cells were plated in 12-well plates containing glass coverslips at 150,000 cells/well density. Twenty-four h after plating, the cells were transfected using the FuGENE^®^ HD Transfection Reagent (Promega Corporation, Madison, WI, USA), according to the manufacturer’s instructions, with 1 μg of the plasmid pCMV6-ENTRY containing the fusion construct HA-Bace1-mBFP [36], 3 μL of FuGENE^®^ HD in Opti-MEM (ThermoFisher Scientific, Waltham, MA, USA) for 24–48 h in a 5% CO_2_ humidified atmosphere at 37 °C. 

To test free antiHA interaction with surface exposing HA fragment, 200 μL of antibodies solution 1 μg/mL (dilution 1:1000 from the stock solution of 1 mg/mL) were added to the transfected cells culture. Then, NSts bioconjugate were incubated as described in Section 2.3. 

### 2.5. Characterization Techniques

The plasmonic properties of gold NPs colloidal solution before and after functionalization processes were acquired in the range from 400 nm to 900 nm with a UV-vis-NIR spectrophotometer (Lambda 950 instrument, Perkin Elmer, Waltham, MA, USA). UV WinLab Software (Perkin Elmer, Waltham, MA, USA) was used to acquire spectra and data were processed with Origin software. The hydrodynamic dimensions, the polydispersity, and the zeta potential were characterized by Dynamic Light Scattering (DLS) analysis performed with a Malvern Zetasizer Nano series ZS90 (Malvern, Worcestershire, UK). Measurements were performed with a fixed scattering angle of 90°, at 25 °C. Each sample was measured three times and each measurement consisted of about 30 acquisitions. Cumulating statistics were used to measure the hydrodynamic diameter and polydispersity. In ζ-potential, each sample was measured three times and each measurement consisted of 100 acquisitions. Data were then processed with Origin software. The NPs morphology in terms of size and shape was characterized by transmission electron microscope (TEM, CM 12 PHILIPS, Amsterdam, The Netherlands).

A horizontal agarose gel system was used under a constant voltage of 150 V in a mini-sub cell GT (Bio-Rad) with agarose from UltraPure^TM^ Agarose, Invitrogen (Waltham, MA, USA) including 0.5% and 0.3% respectively for NSps and NSts in Tris-Acetate-EDTA (TAE) buffer 0.125×. Samples were centrifuged following conditions reported in Section 2.2 and supernatant was discarded. Furthermore, 15 µL of PBS buffer and 5 µL of glycerol were used to resuspend the pellet and to increase sample density to improve well deposition. 

The structural features of the NPs were characterized by transmission electron microscopy (TEM, CM 12 PHILIPS).

Fluorescence assay was performed in 96-well plate with a BMG Labtech FLUOStar Optima (Ortenberg, Germany) microplate reader. Measurements were performed 10 times at 18 °C and gain was set at 1027. All the collected data were then analyzed using Origin software.

Living SH-SY5Y cells transfected with the construct HA-Bace1-mBFP were separately incubated with DyLight550-labelled anti-HA, NSts-PEG, and DyLight550-labelled antiHA-NSts for 30 min at room temperature. The analysis of mBFP and DyLight550 fluorescences was performed after excitation at 405 nm and 561 nm, respectively, using a Nikon C2 laser scanning confocal microscope (Leica, Wetzlar, Germany) and a Plan Fluor 100 × 1.49 NA oil immersion objective. A series of optical sections (1024 × 1024 pixels) at the cell median planes was taken and analyzed using ImageJ software. All settings, including pinhole diameter, detector gain and laser power, were kept constant for each analysis.

Raman spectra were collected with a conventional micro-Raman setup (XploRA PLUS Confocal Raman Microscope, Horiba, Kyoto, Japan), consisting of a 785 nm laser (Coherent, Santa Clara, CA, USA) and a spectrometer with a focal length of 500 mm, equipped with a 600 lines/mm grating. The incident laser power on the sample was about 20 mW. The scattered light was detected by a CCD camera operating at about 350 K. Raman-SERS spectra were recorded in the wavenumber range of 900–1800 cm^−1^, the acquisition time was 5 s and the measurement were repeated ten times for spectral averaging. To avoid spurious signals, calcium fluoride Raman slides (CaF_2_, Crystran, Dorset, UK) were used as substrates. In order to extract the Raman signal of interest, fluorescence and background signals were subtracted from the acquired raw spectra through Vancouver Raman Algorithm, a dedicated software for automatic autofluorescence back-ground subtraction for Raman spectroscopy [37]. Data were further analyzed with Origin software.

## 3. Results and Discussion

**Figure 1:**
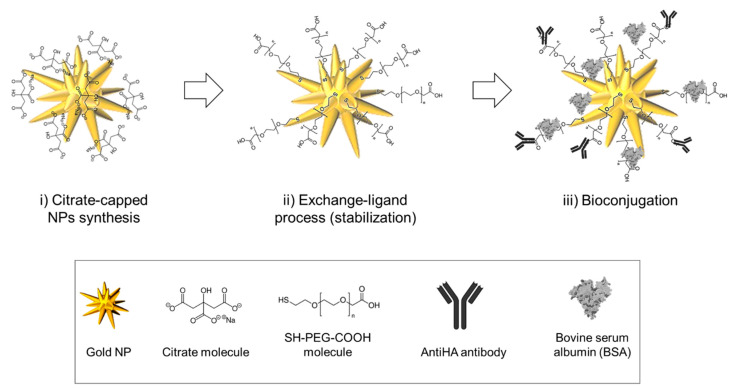
Schematic diagram representing the three-steps functionalization process to antibody-conjugate gold nanoparticles.

Herein, a simple bottom-up process for the construction of an optimized and versatile NPs-bioconjugate is presented. The scheme reported in Figure 1 shows the three steps involved in the functionalization strategy: (i) synthesis of gold nanoparticles, both citrate-stabilized nanospheres and nanostars; (ii) ligand exchange process from citrate to SH-PEG-COOH in order to increase the stability; (iii) bioconjugation with antiHA antibodies for detection and BSA for blocking unspecific adsorption to increase the specificity of the construct.

### 3.1. Assembly of NP-Bioconjugate and Characterization

**Figure 2:**
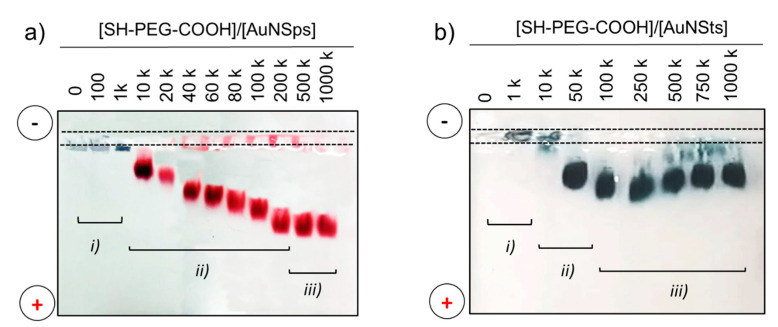
Representative pictures of an agarose gel with (**a**) gold nanospheres (AuNSps) and (**b**) gold nanostars (AuNSts) samples functionalized with increasing molar ratios of SH-PEG-COOH.

First, NPs were produced following the method reported in Section 2.2. Immediately after the synthesis, both spheres- and stars- shaped particles resulted stabilized in an electrostatic way by citrate molecules. Following recent results obtained by our group [38], an exchange ligand process with a bifunctional molecule was implemented in order to raise the stability of the colloidal solution and to simultaneously introduce new functional chemical groups to be further conjugated [25]. Polyethylene glycol (PEG) molecules are the most widely exploited class of polymer to achieve resistance against protein adsorption, enhancing their biocompatibility, and to prevent their aggregation in biological environments. In our case, a bifunctional PEG was anchored to the particle exploiting thiol groups (SH) and exposing carboxyl pendant groups (COOH) exploited for antibody covalent coupling, while inducing an extra stabilization through steric interactions between aliphatic chains. As a matter of fact, functionalization of gold NSps with PEG molecules is a well-known and characterized procedure, the surface engineering of NSts is instead more challenging [21,26,27,28]. Consequently, the exchange ligand process from citrate to PEG was conducted in parallel for both types of particles. At first, different concentration ratios were exploited for ligand exchange and the quantity of SH-PEG-COOH added to the solution of NPs was optimized to achieve the 100% coverage. The optimized amount of SH-PEG-COOH necessary to fully cover the NPs was tested by running agarose gel electrophoresis (AGE), a technique which has found application also in the field of colloid and NPs characterization to find out the modifications in charge and dimensions and consequently to demonstrate functionalization and conjugation processes [14]. NPs are differentiated according to variances in size and surface charge and bands can be discovered without any dye molecules, since NPs solutions are coloured (red and blue for NSps and NSts respectively) due to their plasmonic activities. Figure 2 reports an AGE for NSps (a) and NSts (b) samples functionalized with different molar ratio of SH-PEG-COOH. The quantity of stabilizing agent increases from left to right. Three types of bands are observed: (i) PEG/NPs ratios of 0 and 1 k, corresponding to aggregated AuNPs, which did not move in the gel; (ii) bands at intermediate ratio values (ranging from 10 k to 250 k for NSps and from 10 k to 50 k for NSts), presenting an electrophoretic mobility consistent with a PEG layer partially enveloping the NPs; and (iii) bands presenting the highest mobility, assessing the formation of a full PEG monolayer on gold surface. In both cases, the variation of electrophoretic mobility corresponded to the increase in the net negative charge on the NPs surfaces due to the increase in carboxyl groups content deriving from PEG molecules grafting. The constant trend at ratios higher than 500 k for NSps and 100 k for NSts indicated that surface saturation was achieved corresponding to a full PEG monolayer on the gold surface. Also, the bands at a lower PEG to AuNP molar ratio presented great aggregation and smearing. This effect testifies to the increase in colloidal stability of PEG-NP compared to citrate-NP in PBS 1× solution (0.1 M, ionic strength of 162.7 mM): desorption of citrate molecules, weakly interacting with the gold surface due to the higher ionic strength, induced attraction forces between particles to prevail thus leading to aggregation phenomena. Hence, molar ratios of 500 k for NSps and 100 k for NSts were selected for functionalization parameters as they resulted the minimum concentration required to obtain a full corona at the gold surfaces. The least amount of PEG molecules involved for NSts could be ascribed to the different features of nanoconstructs affecting PEG orientation: in the presence of sphere-shaped particles, PEG aliphatic backbones are probably radially oriented promoting the formation of a close packaging between molecules. Instead, with more complex surface curvature characterizing NSts, PEG chains are randomly orientated, thus possibly increasing the steric repulsions among PEG molecules themselves.

**Figure 3:**
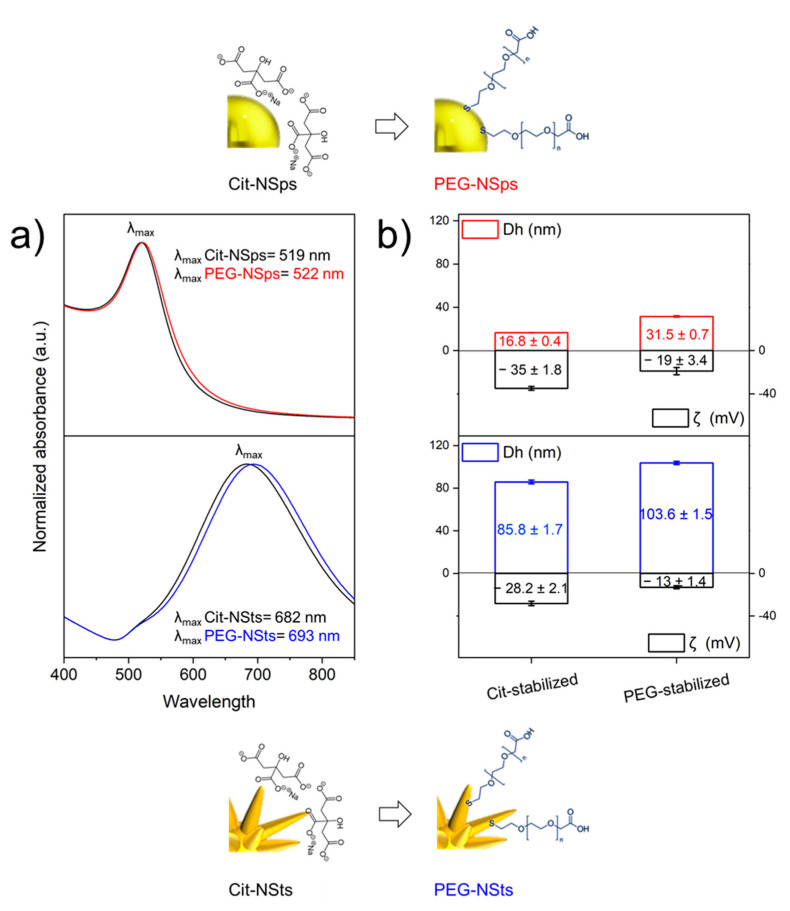
Normalized extinction spectra of gold NSps and NSts colloidal solutions before and after the ligand exchange process (**a**). Size distribution and zeta potential values for citrate- and polyethlyne glycol (PEG)-capped NSps and NSts (**b**).

Optical and physical properties of optimal 500 k and 100 k PEG-NPs systems were characterized. The UV-Vis spectra reported in Figure 3 showed a little bathochromic shift for PEG-NPs respect to Cit-NPs for both spheres and stars. This was the consequence of the increase in the extinction coefficient and therefore in the maximum absorbance wavelength, due to the change of chemical environment at NPs surface after ligand exchange. Calculated Δλ was equal to 3 nm for NSps and to 11 nm for NSts: the larger shift for star-shaped particles was ascribed to the higher sensitivity in the change of local refractive index due to the presence of anisotropic tips or edges, which enhance electromagnetic fields more than spheres-core plasmons [39]. Despite the complex morphology of NSts, whose high aspect ratio tips could have affected the homogeneity of the exchange process, the extinction spectra demonstrated that we successfully accomplished covalent bifunctional-PEG anchoring for NSts. As expected, the size of Cit-NSps was 16.8 ± 0.4 nm and resulted in a slight increase to 31.5 ± 0.7 nm after PEG molecules grafting (Figure 3b). The same trend of 15-nm increase in hydrodynamic dimension upon ligand exchange was measured for NSts with values increasing from 85.8 ± 1.7 nm to 103.6 ± 1.6 nm for Cit-NSts and PEG-NSts, respectively (Figure 3c). Furthermore, zeta potential (ζ) measurements confirmed that citrate to carboxylate SH-PEG replacement was successfully obtained, with ζ decreasing from −35 ± 1.8 mV for citrate to −19 ± 3.4 mV for SH-PEG-COOH for sphere-shaped colloidal solution; the surface charge of NSts experienced a decrease from −28.2 ± 2.1 mV for citrate to −13 ± 1.4 mV for SH-PEG-COOH. Despite the fact that the citrate molecule is characterized by three negatively-charged carboxylic groups against the only one pending from a PEG molecule, the ζ significant decrease when introducing PEG has led to the hypothesis that the higher PEG steric hindrance, with respect to citrate, affects molecules loading on AuNSts, thus resulting in lower grafting density. Moreover, the higher negative value of ζ characterizing PEG-NSps, with respect to PEG-NSts, suggests a lower number of negatively-charged stabilizing molecules due to the different particles morphology, confirming results obtained with AGE (500 k molar ratio needed for stabilize NSps vs. 100 k for fully cover NSts).

Finally, the best optical performances of NSts, with respect to NSps, as a direct consequence of the combination of inherent anisotropic shapes and exceptional optical functionalities were experimentally demonstrated in our previous work [40]. Henceforth, discussion was focused on NSts to demonstrate the feasibility of a covalent graft antibody-conjugation process.

### 3.2. Optimization and Quantification Bioconjugation Process (Ab-NSts)

**Figure 4:**
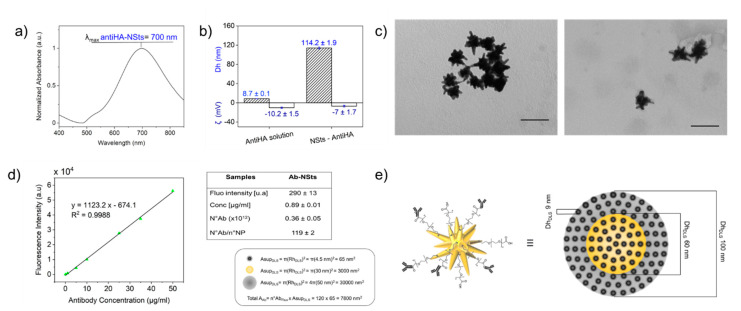
Normalized extinction spectra of gold nanostars colloidal solutions after antibody-conjugation (**a**). Size distribution and zeta potential values for free antiHA in solution and antiHA-conjugated NSts (**b**). TEM images of Cit-NSts and Ab-NSts, respectively. Scale bar is 100 nm in all cases (**c**). Calibration curve to quantify the number of Abs conjugated to the nanoparticle (NP), derived from the fluorescence intensities of standard solutions of Alexa488-labelled IgG antibodies; table resuming main results for NSts-Ab bioconjugate (**d**). Graphical model representing the NP spherical surface homogeneously covered by the area of the antibodies (**e**).

Antihemagglutinin antibodies (antiHA) were grafted onto a PEG-modified gold surface, by coupling carboxyl groups of PEG-stabilized NSts to primary amines present in the amino acid side chains of the Abs using standardized methods, e.g., EDC/NHS activation. A 10-nm red-shift of the LSPR was distinguished upon functionalization with antiHA (see Figure 4a). DLS measurements confirmed that NSts-Ab complexes were stable (polydispersity index changes from 0.30 ± 0.01 to 0.32 ± 0.02) and a 10 nm increasing of the hydrodynamic radius compared to bare NSts attested to the successful bioconjugation of Abs, whose diameter measured with DLS is about 9 nm (Figure 4b). The zeta potential of antiHA in PBS buffer was −10.2 ± 1.5 mV; the net charge of NSts functionalized with antiHA was anticipated to be not far from that of the protein itself, if the protein covered the surface of AuNP. ζ of bioconjugate shifted from −13 ± 1.4 mV with PEG to −7 ± 1.7 mV, in agreement with the ζ value measured for antibodies (Figure 4b). TEM images (Figure 4c) revealed that the NSts has a large number of sharp protruding tips, which were retained upon functionalization with both PEG stabilizing molecules and antibodies, thereby ensuring retention of their morphology-dependent SERS activity.

Cross-linkage between Ab and PEG stabilized-NSts can be further detected and quantified by fluorescence measurements exploiting an Alexa488-labelled IgG immunoglobulin. It is worth noting that in our case a direct quantification of fluorophores attached to NPs was possible because fluorescence signal is quenched in the very close proximity (less than 5 nm) or through direct contact with the metal surface. Therefore, standard solutions of Alexa488-labelled IgG antibodies were used to build the calibration curve and to quantify the number of Abs conjugated to each NP (Figure 4d). After the functionalization process, the solution containing bioconjugate was washed to remove non-reacted molecules. Low rpm and short time of centrifuge were exploited to separate between Ab-NSts constructs and free Ab small molecules, which should remain in the supernatant. Fluorescence intensities were collected from both pellets containing functionalized NSts and supernatant including unreacted antibodies. Table in Table S1 (see ESI) reports theoretical calculations of the number of molecules of immunoglobulin through estimation of mol. It results that the number of antibodies in the system is 8 × 10^12^, which means 4000 Ab attached per particle. Experimental values based on collected fluorescence intensities for NSts-Ab bioconjugate are presented in Figure 4d. Consequently, the concentration of labelled antibodies were derived by means of line equation fitting the calibration curve. It results that the number of antibodies conjugated to NSts was about (0.36 ± 0.05) × 10^12^ or 0.89 ± 0.01 µg/mL in terms of concentration, resulting in 119 ± 2 antibodies per particle. (6.88 ± 0.01) × 10^12^ unbound antibodies resulted in the supernatant (table Table S1 in ESI), with a concentration of 17.59 ± 0.92 µg/mL. Partial loss of Ab in the amount of about 1 µg/mL, was probably due to adsorption occurring at the sample vial surface or some aggregation phenomena. However, loading of about 120 Abs per particle are consistent with previous work implemented on gold nanospheres and the lower Ab coverage in comparison with NSps could be attributed to a shape effect [7,41]. 

By combining DLS and fluorescence data, the total surface area occupied by the antibodies (Total A_ab_) (n°Abs calculated by fluorescence measurements multiplied for the area derived from the hydrodynamic radius (Rh)) was compared with surface area of the sphere built considering Rh of the NSts (Asup_DLS_). As it could be expected, Total A_ab_ < Asup_DLS_. The data fit well a model in which an homogeneous distribution of slightly spaced linked Abs cover the entire approximated spherical surface of the NSts, as graphically schematized in Figure 4e (where the graphical scheme is designed to keep the proportions between the objects).

Since the successfully reported functionalization strategy involves amide bond formation between carboxyl functionalities attached onto gold surface and amine groups of antibodies, it is worth noting that the same process can be exploited for grafting diverse ligands for biomolecular identification, characterized by the same chemical reactivity. In other words, every type of immunoglobulin G could be conjugated to PEG-modified NPs through this optimized strategy, leading to great versatility in terms of possible diagnostics as well as therapeutics applications.

### 3.3. In Vitro Test of Activity and Selectivity of Bioconjugates

In vitro experiments were performed to assess whether the specificity of the antibody was preserved after covalent grafting. To this end, antiHA conjugated NPs were incubated with human SH-SY5Y cells transfected with an engineered protein construct, that was designed to express Bace1, a transmembrane protein, fused to mBFP at the C-terminus and to HA at the N-terminus (HA-Bace1-mBFP) [36]. Once the overexpressed fusion protein reaches the plasma membrane, the N-terminal domain carrying the HA tag is exposed on the extracellular side of the cell; this latter represents the target for the DyLight550-labelled antiHA-NSts. Since the transfection efficiency was about 20–25%, the idea conceived that fluorescent antiHA-NSts would surface label only HA-Bace1-mBFP expressing cells, resulting in colocalization of fluorescence signals of mBFP and DyLight550. 

**Figure 5:**
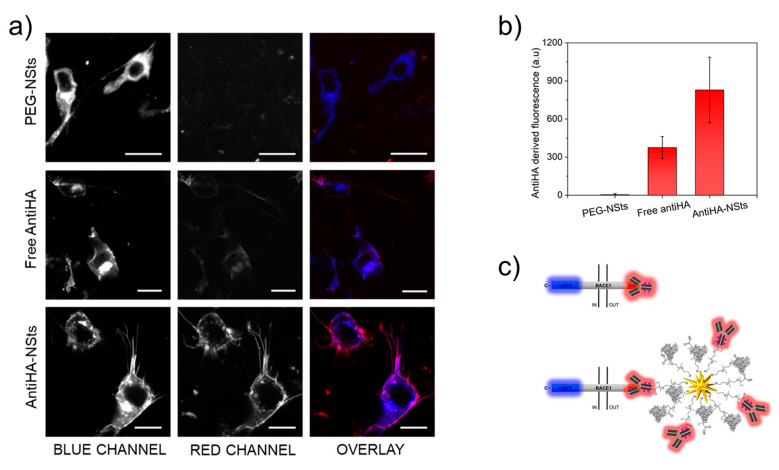
Representative confocal microscope images of SH-SY5Y cells transfected with the HA-Bace1-mBFP fusion construct and incubated with PEG-NSts, free antiHA and antiHA-NSts. Scale bar is 10 μm in all cases. Blue and red fluorescences indicate mBFP and antiHA, respectively. The images of 20 cells were analyzed at median planes parallel to the coverslip using ImageJ software (**a**). Histogram showing the quantitative values of antiHA fluorescence measured for the three sample types after background subtraction. Error bars are SD (**b**). Schematic representation of the process of peptide targeting by both free antiHA and multivalent antiHA-NSts bioconjugate (**c**).

Confocal fluorescence microscopy images were acquired during the in vitro experiments for transfected cells separately incubated with DyLight550-labelled antiHA, named as PEG-NSts as negative control (row 1), free antiHA (row 2), and DyLight550-labelled antiHA-NSts (row 3) (Figure 5a). The images show the mBFP fluorescence corresponding to the transfected cells (blue channel, column 1), the DyLight550-labelled antiHA fluorescence, in the absence and in the presence of NSts (red channel, column 2) and the merge of the two channels for each type of sample incubation (column 3). To enable signal comparison based on fluorescence intensities, the same theoretical number of Ab molecules was exploited for NPs functionalization process (antiHA-NSts) and for cells incubated with free antiHA. The fluorescence signal from the blue channel was clearly observed for all the three incubations, thus confirming that cell transfection was successfully achieved. On the other hand, the red signal was observed only when cells were incubated in the face of antiHA-NSts, with total colocalization of the blue and red signals (merge channel), thus demonstrating the high tag selectivity and specificity of the NPs constructs even in the presence of samples as highly heterogenous as cell cultures. Furthermore, the fluorescence was strictly localized as a “membrane surrounding corona” on the surface of cells, suggesting that the NPs remained associated with the cell membrane and are not taken into the cell within the 30-min incubation period. No aspecific red signal was observed in cell not expressing HA-Bace1-mBFP, which is a clear demonstration of the high colloidal solution stability (PEG-functionality) when in the presence of high ionic strength solutions. In the control sample, represented by cells incubated with bare NSts-PEG, a very low red fluorescence signal, very close to the fluorescence values of the background, was observed. Moreover, biocompatibility of the engineered construct was indirectly tested through observation of the viability of cells, which was preserved since no visible changes in the cell morphology, as well as in the number of cells on the coverslip, were detected over the experimental time. 

It is also important to highlight the difference in intensity of the signal of unconjugated antibodies (free antiHA) with respect to antiHA-NSts. Actually, the fluorescence intensity after background subtraction reported in Figure 5b resulted to be three times higher for antiHA-NSts samples with respect to free antiHA samples. This was due to the multivalency properties characterizing functionalized NPs, where the surface of one nanoparticle can bind up to 120 antibodies as schematized in Figure 5c and previously demonstrated in Section 3.3. Consequently, although the same amount of Ab was used for “free” and “NP-grafted” cells incubation, in “free” samples tests each Bace1 site links to one antiHA, while in “NP-grafted” samples tests each Bace1 site links to a 120 antiHA-complex (one directly coupled to the Bace1 site and the other 119 grafted to the NP).

The multivalency could be exploited to design more efficacious diagnostic agents, as, for example, enhancers of optical transduction signal in the sensoristic field [42]. In our case, the possibility to tie multiple analytes for each particle was exploited for the development of SERS-substrates for the analysis of biofluids, by taking advantage of the interaction between gold enhancing surface and more baits at the same time, leading to a huge increase in optical signal. Moreover, it is important to stress once again how the accomplished sensor presents great versatility in terms of analyte to be detected, simply by changing (bio)molecules to be attached to NPs, as a recognition system. 

### 3.4. SERS Activity of Bioconjugates

**Figure 6:**
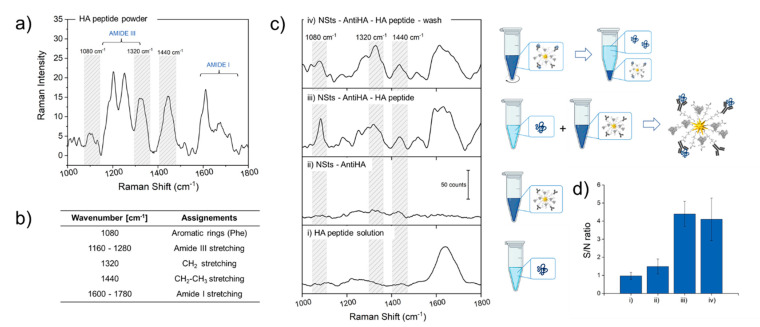
Raman spectra performed on HA peptide powder (**a**), peaks identification and assignment (**b**). Raman spectra of (i) hemagglutinin protein in PBS solution, (ii) AntiHA-NSts bioconjugates in PBS solution, antiHA-NSts interacting with HA peptide after (iii) and (iv) before centrifugation. Spectra have been shifted vertically for clarity of presentation (**c**). Signal to noise ratio with standard deviations calculated for three different peaks (1078, 1283, and 1327 cm^−1^) of the four different samples (**d**).

To demonstrate the potentiality of developed bioconjugates to work as SERS-based substrates for the detection of analytes diluted in fluids, antiHA-NSts were incubated with an aqueous solution of 500 µM HA peptide and Raman spectra were collected. Raman spectra for HA peptide lyophilized powder was acquired to identify characteristic peaks (Figure 6a) and main stretching/bending modes were assigned and reported in table in Figure 6b, while spectra of NSts-Ab were also recorded and reported in Figure 6c to be taken as reference. The Raman spectra of protein contain lots of chemical information deriving mainly from three types of contributes: the polypeptide backbones contributing to the amide bands, and both the aromatic and non-aromatic amino acid side chain residues. The amide III band (1160–1280 cm^−1^) pronounced in solid powder is not even visible in the Raman spectra of HA solution, while the intensity of the signal is enhanced in presence of metallic nanostructures. The same trend is recognized also for amide I band (1600–1780 cm^−1^), which is primarily related to C–N stretching, C–C stretching, and N–H bending. Predominant peaks in the HA powder spectrum at 1080 cm^−1^, produced by the C–C and C–N stretching of phenylalanine, 1320 cm^−1^ peak due to CH_2_ stretching and 1440 cm^−1^ (CH_2_ –CH_3_ stretching) are not visible in HA protein in liquid buffer. Raman intensity of characteristic peaks is 4-fold enhanced by NSts-Ab substrate. Quantification of signal increasing is given by comparing signal to noise ratio mediated on three different peaks (1080, 1320 and 1440 cm^−1^) of Raman spectra of the different samples (Figure 6c), graphically represented in the histogram in Figure 6d with their standard deviation. As expected, characteristic Raman spectra of HA peptide were obtained only in the face of NSts-Ab, thanks to the presence of nanostructured metallic surface, characterized by geometrical characteristics showing sharp tips that localize and amplify the approaching electromagnetic radiation (the so-called “hot-spots”) as well as to high near-infrared absorbance. Despite the high tag selectivity previously demonstrated with in vitro experiments, to further test if Raman enhancement in the presence of NSts was not due to aspecific HA adsorption or to random HA localization nearby the NPs, the solution containing bioconjugates and HA peptide was further centrifuged and the Raman spectrum was recorded again. The intermediate centrifuge step was exploited to separate by precipitation covalently bind HA-NPs conjugates. If peptide was not housed in its specific binding site, during the washing step it would remain in solution in the supernatant, while NSts-antibody conjugated would precipitate for gravity. Zeta potential values reported in Figure 4b show that, at the working pH values of 7.2–7.4, both HA peptide and NSts bioconjugate are negatively charged and so interactions between NSts-antibody and HA protein are thus not electrostatically driven, but involve strongly antigen-antibody interaction. Raman spectra after and before centrifuge (wash) in Figure 6c presented the same highlighted principal bands, confirming our hypothesis. 

## 4. Conclusions

The conjugation of several moieties to the NPs widens their employment fields and supplies them with new or enhanced properties. In the present work, we report a successful and simple bottom-up process for the construction of NPs- bioconjugate, characterized by high particle stability, selectivity, and versatility in terms of biological target, to be used for SERS detection. In particular, we focused our attention on the design and optimization of the bottom-up process for increasing AuNSts stabilization in high ionic strength buffer, covalent conjugation with antibodies while retaining the specific biofunctionality to specific tag analytes within the fluid. To this end, an exchange ligand process with PEG bifunctional molecules was optimized to completely cover the surface of the NPs in order to increase the stability of the colloidal solution. Then, carboxyl pending groups immobilized onto gold nanostars’ surface through PEG were further bioconjugated with baits specifically for recognition of the target analyte. The construct was intensively characterized to assess the efficiency of implemented process. Then, the retained specificity of the antibody was tested against cells overexpressing the antigen of interest. Moreover, potentiality of developed bioconjugates as SERS-based substrates for detection of analytes diluted in fluids were demonstrated with Raman measurements performed against HA peptide diluted in aqueous solution. Finally, the present work should be considered as a background study for the development of cheap, easy, reliable, rapid, and versatile plasmonic-based optical sensors, useful for testing liquid samples and in particular biological fluids. We see a great potential in further developing tunable SERS-substrates by taking advantage of different baits to be attached onto NPs surface, allowing great versatility in terms of analyte to be detected.

## Data Availability

The data presented in this study are available on request from the corresponding author.

## Supplementary Materials

The following are available online at https://www.mdpi.com/2079-4991/11/3/665/s1, Table S1: Table resuming main results for Ab solution of 20 µg/mL, which is the quantity used to functionalized NSts, as direct control and surnatant of Ab-NSts after centrifuge.
