# Supplementary material for: Gold Nanostars Bioconjugation for Selective Targeting and SERS Detection of Biofluids
Source: Nanomaterials (Basel). 2021 Mar 8;11(3):665. doi: 10.3390/nano11030665 (PMC8000610; doi:10.3390/nano11030665)
Supplement: Supplementary file 1 [file nanomaterials-11-00665-s001.pdf]

nanomaterials

MDPI

Supplementary Material

Gold Nanostars Bioconjugation for Selective Targeting and SERS Detection of Biofluids

Caterina Dallari [1,*], Claudia Capitini [1,2], Martino Calamai [1,3], Andrea Trabocchi [4], Francesco Saverio Pavone [1,2,3] and Caterina Credi [1,3,*]

[1] European Laboratory for non-linear Spectroscopy (LENS), University of Florence, 50019 Sesto Fiorentino, Florence, Italy; capitini@lens.unifi.it (C.C.); calamai@lens.unifi.it (M.C.); francesco.pavone@unifi.it (F.S.P.)
[2] Department of Physics, University of Florence, 50019 Sesto Fiorentino, Florence, Italy
[3] National Institute of Optics -National Research Council (CNR-INO), 50019 Sesto Fiorentino, Florence, Italy
[4] Department of Chemistry "Ugo Schiff", University of Florence, 50019 Sesto Fiorentino, Florence, Italy; andrea.trabocchi@unifi.it (A.T.)
* Correspondence: dallari@lens.unifi.it (C.D.); credi@lens.unifi.it (C.C.)

Table S1. Table resuming main results for Ab solution of 20 µg/mL, which is the quantity used to functionalized NSts, as direct control and surnatant of Ab-NSts after centrifuge.

Samples	20 µg/mL	Surnatant
Fluo intensity [a.u]	-	14635 ± 147
Conc [µg/mL]	20	17.59 ± 0.92
N°Ab (×10^{12})	8	6.88 ± 0.01
N°Ab/n°NP	4000	-
